# Common Activation of Canonical Wnt Signaling in Pancreatic Adenocarcinoma

**DOI:** 10.1371/journal.pone.0001155

**Published:** 2007-11-07

**Authors:** Marina Pasca di Magliano, Andrew V. Biankin, Patrick W. Heiser, David A. Cano, Pedro J. A. Gutierrez, Therese Deramaudt, Davendra Segara, Amanda C. Dawson, James G. Kench, Susan M. Henshall, Robert L. Sutherland, Andrzej Dlugosz, Anil K. Rustgi, Matthias Hebrok

**Affiliations:** 1 Diabetes Center, Department of Medicine, University of California at San Francisco, San Francisco, California, United States of America; 2 Cancer Research Program, Garvan Institute of Medical Research, St. Vincent's Hospital Campus, Darlinghurst, New South Wales, Australia; 3 Department of Surgery, Bankstown Hospital, Sydney, Australia; 4 Gastroenterology Division, Department of Genetics, University of Pennsylvania, Philadelphia, Pennsylvania, United States of America; 5 Comprehensive Cancer Center, Department of Dermatology, University of Michigan, Ann Arbor, Michigan, United States of America; RIKEN Genomic Sciences Center, Japan

## Abstract

Pancreatic ductal adenocarcinoma (PDA) is an extremely aggressive malignancy, which carries a dismal prognosis. Activating mutations of the Kras gene are common to the vast majority of human PDA. In addition, recent studies have demonstrated that embryonic signaling pathway such as Hedgehog and Notch are inappropriately upregulated in this disease. The role of another embryonic signaling pathway, namely the canonical Wnt cascade, is still controversial. Here, we use gene array analysis as a platform to demonstrate general activation of the canonical arm of the Wnt pathway in human PDA. Furthermore, we provide evidence for Wnt activation in mouse models of pancreatic cancer. Our results also indicate that Wnt signaling might be activated downstream of Hedgehog signaling, which is an early event in PDA evolution. Wnt inhibition blocked proliferation and induced apoptosis of cultured adenocarcinoma cells, thereby providing evidence to support the development of novel therapeutical strategies for Wnt inhibition in pancreatic adenocarcinoma.

## Introduction

Pancreatic ductal adenocarcinoma (PDA) is the fourth leading cause of cancer death in Western societies. The disease usually presents at an advanced stage, and as a result only 10 to 20% of patients are suitable for surgical resection [1]. Presently, non-operative therapies are widely ineffective, contributing to an overall 5 year survival-rate of less than 5%. There is now compelling histopathological and molecular evidence to support the evolution of PDA through a series of non-invasive duct lesions called pancreatic intraepithelial neoplasia (PanIN) [2]. Progression of PanIN lesion is associated with molecular aberrations that increase in frequency and correlate with advancing cellular atypia from early stages to invasive cancer [3], [4]. Recent studies have identified deregulation of pathways important in vertebrate pancreas development, including Notch [5] and Hedgehog [6], [7], in the development and progression of PDA.

Interactions between embryonic signaling pathways ensure proper organ formation during development. Increasing evidence suggests that these pathways remain active in a subset of cells within adult organs and that deregulation of their activity contributes to the development and progression of certain tumors [8]. Hedgehog and Wnt signaling are involved in the development of the pancreas [9]–[13]. Both pathways appear to be regulated in a very tight manner during embryogenesis. Inappropriate activation of Hedgehog signaling during pancreas formation results in agenesis of this organ [14], [15]. A similar result is observed when Wnt signaling is activated at high levels during early pancreatic development [10], [16]. In contrast, activation of Wnt signaling predominately in acinar cells results in a significant increase in pancreas mass [10]. Ectopic activation of Wnt signaling at early stages of pancreas organogenesis increases Hedgehog activity [10]. While Hedgehog signaling is known to regulate Wnt activity in other organs [17], such regulation in developing pancreatic tissue has not yet been reported.

A large body of evidence supports the notion that uncontrolled activation of the canonical Wnt pathway induces tumor formation in the distal gastrointestinal tract [18]. Canonical Wnt signaling is activated when soluble Wnt ligands form a complex with one of several FRIZZLED receptors and LRP5/LRP6 co-receptors. This interaction triggers a cascade of events that results in the inhibition of ß-CATENIN phosphorylation. Non-phosphorylated ß-CATENIN translocates from the cytoplasm to the nucleus where it binds to the TCF-LEF family of transcription factors to activate the transcription of Wnt target genes. Upregulation of Wnt signaling, mediated by specific mutations in the APC or ß-CATENIN genes, is thought to play a critical role in the development of several gastrointestinal tumors [18], [19].

Sustained tumor growth due to deregulation of Wnt signaling independent of mutations that increase pathway activity has been demonstrated in studies on breast and ovarian cancer [20]. Similarly, mutations in either APC or ß-CATENIN that are commonly found in other gastrointestinal cancer are rare in PDA [21]. However, aberrant cytoplasmic and nuclear expression of ß-CATENIN, both indicative of canonical Wnt signaling activity [11], , are present in a substantial group of PDA and PanIN samples [23], [24]. Moreover, heparan sulfate proteoglycans, known to regulate Wnt activity, are expressed in pancreatic adenocarcinoma and positively regulate cancer growth [25]. These data suggest that Wnt signaling may play a role in PDA despite the absence of signature mutations in APC or ß-CATENIN. Nevertheless, activation of the Wnt signaling pathway in pancreatic cancer has remained controversial [24] and functional studies addressing a potential contribution of Wnt signaling to PDA development and progression are currently missing.

Here we identify aberrant expression of Wnt signaling components in a large cohort of patients with PDA. Whilst only 13% of PDA demonstrate nuclear localization of ß-CATENIN, 65% demonstrate either loss of membranous expression and/or increased cytoplasmic expression. Similar results were obtained from the analysis of mouse models of pancreatic cancer. Assessment of PDA cell lines revealed activation of Wnt signaling and pathway inhibition showed that cancer cell survival and proliferation depends in part on Wnt activity. Increased levels of Hedgehog signaling constitute one of the earliest changes in PanIN lesions. Our results show that Hedgehog signaling activates ß–CATENIN/Wnt signaling in transgenic mice and untransformed pancreatic duct cells, suggesting that Hedgehog may play a role in upregulating Wnt activity in PDA.

## Results and Discussion

A number of recent reports have characterized the role of Wnt signaling during pancreas development and function of the adult organ [10]–[13], [26], [27]. In contrast, the exact role canonical Wnt signaling plays in the formation and progression of PDA remains unclear. While robust activation of the pathway due to signature mutations in components of the Wnt cascade commonly observed in other gastrointestinal cancers have not been found in PDA, immunohistochemical analysis against ß-CATENIN, the key mediator of this pathway, suggests a contribution of Wnt signaling during PanIN progression and in PDA [23], [24], [28]. In normal adult pancreatic tissue β−CATENIN is exclusively localized to the cell membrane and both cytoplasmic and nuclear localization of ß-CATENIN is commonly regarded as an indicator of active canonical Wnt signaling [11], [22]. Our analysis of 136 human PDA samples revealed nuclear β-CATENIN expression in 17 (13%) and cytoplasmic expression in 89 (65%) samples (Figure S1). Thus, abnormal localization of β−CATENIN, either nuclear or cytoplasmic, was detected in the majority of the tumor samples.

To obtain more quantitative information about the level of Wnt signaling, we performed expression profiling of Wnt pathway components in PDA with genechip microarray. This analysis demonstrated aberrant expression of numerous components of the Wnt pathway in 12 PDA samples when compared to control tissue (6 normal specimens from the cancer patients and 12 samples of healthy pancreas) (Table S1). Expression of members of the FRIZZLED receptor family (FRIZZLED 2, 7 and 9), their ligands (WNT 2, 3, 4, 5A, 5B, 6, 8B and 11), and putative inhibitors of Wnt signalling (SECRETED FRIZZLED-RELATED PROTEIN 3 and 4 and DICKKOPF genes 1, 2 and 3) was increased in PDA compared to normal pancreas. Central pathway inhibitors such as ICAT were downregulated. In addition, expression of transcriptional target genes of ß-CATENIN, including CYCLIN D1, FIBRONECTIN, RETINOIC ACID RECEPTOR γ, CYCLO-OXYGENASE 2, uPAR and MMP-7, were upregulated. Thus, the overall pattern, particularly the upregulation of direct transcriptional targets of Wnt signaling, suggests increased pathway activity. However, it should be noted that the overall increases in Wnt signaling targets appear smaller than those observed in other tumors, including colon cancer. This might be due to the fact that high level Wnt signaling requires mutations in pathway components, including ß-CATENIN and APC, that are not commonly observed in PDA. This raised the question of whether Wnt signaling is controlled by other signaling pathways known to be deregulated in PanIN and PDA.

### Increased Wnt signaling in a mouse model of PDA

**Figure 1:**
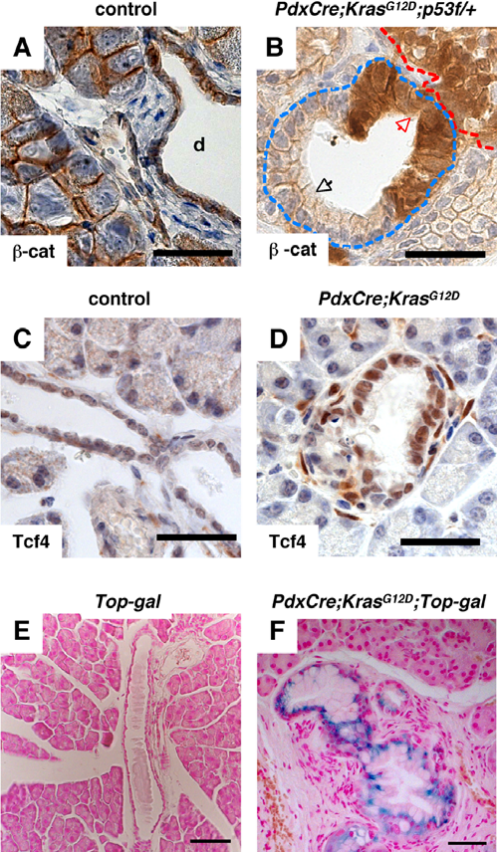
Active Wnt signaling in a mouse model of PDA. A. Immunostaning for β-Catenin in a control pancreas is confined to the plasma membrane. B. In contrast, cytoplasmic β-catenin expression is observed in PanIN lesions and tumor of a 8-week old *Pdx-Cre;Kras^G12D^;p53^f/+^* pancreas. The tumor is outlined in red. Note the accumulation of β-catenin in the epithelial cells (brown staining). Two PanIN lesions are outlined in light blue. The black arrow points at cells that have retained membranous β-catenin, while the red arrows point at areas of accumulation of β-catenin in the cytoplasm. C. Tcf4 is expressed at a low level in ductal cells in a wild-type mouse pancreas. D. Elevated expression of Tcf4 in PanIN lesions of a 12-week old *Pdx-Cre;Kras^G12D^* pancreas. E, F. LacZ staining of pancreatic tissue of adult (6 months old) *Top-gal* (E) and *Pdx-Cre;Kras^G12D^;Top-gal* (F) mice. No staining is present in the *Top-gal* pancreas. ß-galactosidase activity is detected in the PanIN lesions of the *Pdx-Cre;Kras^G12D^;Top-gal* pancreas, indicating activation of the canonical Wnt pathway. The black bar represents 35 µm in A-D, 67.5 µm in E, F.

Deregulation of Kras activity is observed in the vast majority of human PDA [1]. Activating mutations in the *Kras* oncogene have been observed in PanIN lesions, suggesting that deregulation of Ras signaling is a key component during the transformation of epithelial cells [29], [30]. Support for this notion comes from studies in *Pdx-Cre;Kras^G12D^* transgenic mice in which expression of the prevalent *Kras^G12D^* mutation in pancreatic epithelial cells results in the formation of tumors that resemble human PDA [31]. To determine whether increased Wnt signaling is a common phenomenon during PDA formation in different species, we examined pancreata from *Pdx-Cre;Kras^G12D^* and *Pdx-Cre;Kras^G12D^;p53^f/+^* mice by immunohistochemistry. In support of the human data, PanIN lesions in *Pdx-Cre;Kras^G12D^* and *Pdx-Cre;Kras^G12D^;p53^f/+^* mice displayed changes in ß-catenin localization and increased expression of Tcf4, a mediator of canonical Wnt signaling (Fig. 1A–D and Table S1). Quantitative PCR for Wnt target genes showed a moderate increase in *axin2* expression in *Pdx-Cre;Kras^G12D^* pancreata marked by PanIN lesions (Fig. S2). In order to determine whether accumulation of β-catenin effectively correlated with Wnt signaling activity we generated *Pdx-Cre;Kras^G12D^;TOPGal* mice. Due to the expression of the LacZ-reporter gene under control of Wnt signaling responsive TCF-binding sites, the *TOPGal* mice provide an excellent means to identify cells within a given tissue that respond to canonical Wnt signaling [32]. While ß-galactosidase staining is absent in pancreata of control mice (wild type, *TOPGal* and *Pdx-Cre;TOPGal* animals were used as negative controls) (Fig. 1E and data not shown), pronounced ß-galactosidase activity is noted in epithelial cells of PanIN lesions of compound *Pdx-Cre;Kras^G12D^;TOPGal* mice (Fig. 1F). Thus, immunohistochemical staining, quantitative PCR for canonical Wnt target genes, and analysis of Wnt reporter mice indicate increased Wnt/ß-catenin signaling during the formation of PDA in human samples and mouse models of this disease.

### The canonical Wnt signaling arm is active in human pancreatic adenocarcinoma

**Figure 2:**
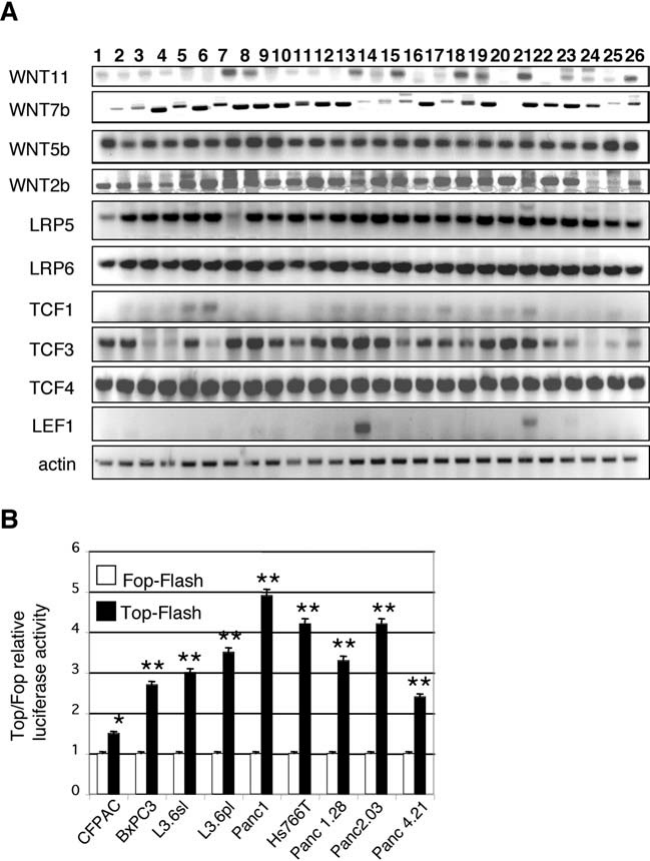
Wnt signaling is active in human pancreatic adenocarcinoma cell lines. A. RT-PCR analysis for components of the Wnt signaling pathway in 26 human pancreatic adenocarcinoma cell lines: 1-MiaPaca2; 2-Panc1; 3-CFPAC1; 4-HPAFII; 5-Capan-2; 6-AsPC1; 7-Hs766T; 8-BxPC3; 9-COLO357; 10-L3.3; 11-L3.6sl; 12-L3.6pl; 13-SW1990; 14-SU86.86; 15-PL45; 16-HPAC; 17-MPanc96; 18-Panc1.28; 19-Panc2.03; 20-Panc2.13; 21-Panc3.27; 22-Panc4.21; 23-Panc5.04; 24-Panc6.03; 25-Panc8.13; 26-Panc10.05. B. Wnt signaling is active in all nine pancreatic adenocarcinoma cell lines tested, as indicated by the activation of the Top-Flash reporter (black) compared to the basal activity of the Fop-Flash reporter (white). Renilla luciferase was used to normalize for transfection efficiency. Error bars are shown as St. Dev. P-values were calculated in comparison to control Fop-Flash activity (white bars). *, p<0.05; **, p<0.01.

To understand whether sustained Wnt signaling is important for cancer cell proliferation we included established pancreatic cancer cell lines in our analysis. First, we used RT-PCR to demonstrate expression of Wnt ligands (*WNT 11, WNT 7b, WNT 5b, WNT 2b*), Wnt ligand co–receptors *LRP5* and *LRP6*, and transcription factors *TCF3* and *TCF4*, in 26 human PDA cell lines derived from either primary or metastatic PDA (Fig. 2A). Expression of *TCF1* and *LEF1*, transcriptional activators of the canonical Wnt signaling arm in other tissues, were expressed only in a subset of cell lines. These data suggest that the proteins necessary for active Wnt signaling are expressed at significant levels in all PDA cell lines analyzed.

To quantitatively evaluate the degree of Wnt signaling activity in a subset of these cell lines (n = 9) we measured luciferase expression controlled by concatemers of ‘TCF optimal sites’ (Top) upstream of a minimal thymidine kinase (TK) promoter element [33]. Concatemers of TCF ‘far from optimal sites’ (Fop) upstream of the TK-promoter served as control for Wnt independent, basal activity of the reporter construct. We detected activation of the Top-Flash reporter in each of the nine different adenocarcinoma cell lines tested (Fig. 2B). The majority of cell lines displayed a 3 to 5 fold relative activation of the Top-FLASH reporter. In comparison, relative Top-FLASH activity in the SW480 and HCT116 colon cancer cell lines was greater than that seen in any PDA cell lines (>6 Top/Fop ratio and >10 Top/Fop ratio respectively, data not shown), indicating that the majority of PDA cell lines show a moderate, but significant increase in Wnt activity.

### Inhibition of Wnt signaling reduces cell proliferation and increases apoptosis in pancreatic adenocarcinoma cells

**Figure 3:**
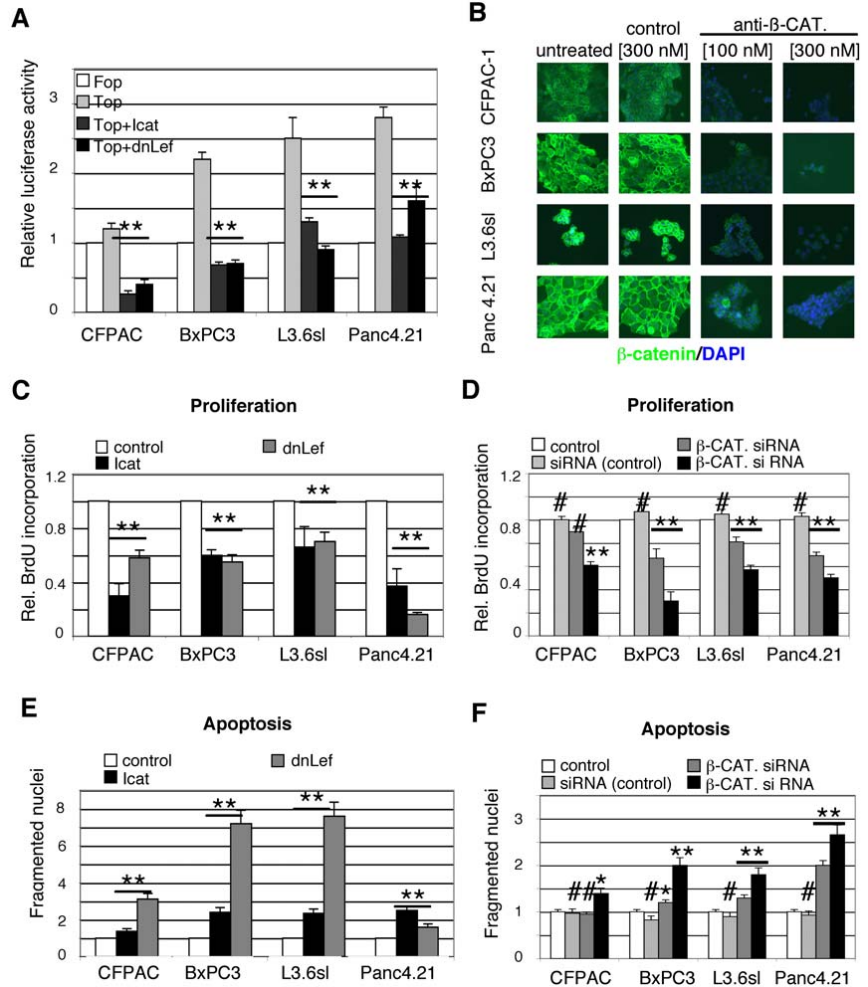
Inhibition of Wnt signaling blocks adenocarcinoma cell proliferation. A. Inhibition of Wnt signaling in four pancreatic adenocarcinoma cell lines. Cells transfected with Fop-Flash (white) or Top-Flash (light gray) reporter constructs were co-transfected with an empty expression vector or an expression vector containing the Wnt-inhibitors *Icat* (dark gray) or dominant-negative *Lef1* (*dnLef*; black). Fop-Flash activity was not affected by either *Icat* or *dn-Lef* co-transfection, thus only one Fop-Flash data point is shown. The significant reduction of Top-Flash activity in cells expressing the inhibitors indicates inhibition of Wnt signaling in pancreatic cancer cells. P-values were calculated in comparison to Top-Flash activity (light gray bar). B. Immunofluorescent staining against β-*CATENIN* in four pancreatic adenocarcinoma cell lines after transfection with a siRNA directed against β-*CATENIN*. The β-CATENIN protein levels are dramatically decreased in a dose-dependent manner following β-*CATENIN* siRNA transfection. Control siRNA transfected cells have β-CATENIN levels indistinguishable from those found in untransfected cells (untreated). C. Transfection with an *Icat–IRES-eGFP* expression vector (*Icat*, black) or dominant negative Lef – *IRES-eGFP* (*dnLef*, gray) strongly inhibits growth of four pancreatic cancer cells lines, measured as the ability to incorporate BrdU. Control cells (white) were transfected with the *IRES-eGFP* expression vector; all cells were harvested 48 hrs after transfection. P-values are shown in comparison to control transfected cells. D. Proliferation is reduced in a dose-dependent manner in cells treated for 48hrs with anti-ß-*CATENIN* siRNA (dark grey, black,) compared to control cells (white). Untreated (no siRNA), white columns; control siRNA (300 nM), light grey columns; anti-ß-*CATENIN* siRNA (100 nM), dark grey columns; anti-ß-*CATENIN* siRNA (300 nM), black columns. P-values are shown in comparison to untreated controls (white columns). E. Level of apoptosis, measured as cells with DNA content lower than the diploid amount, in control transfected cells (white) or cells transfected with *Icat* (black) or dominant negative *Lef* (grey). F. Treatment with anti-ß-*CATENIN* siRNA increases levels of apoptosis in a concentration-dependent manner. Error bars are shown as St. Dev.; P-values, #, not significant; *, p<0.05; **, p<0.01.

Four pancreatic adenocarcinoma cell lines were selected for in depth analysis of the requirement of Wnt signaling in PDA cells. Two well-established lines (CFPAC, BxPC3), a line with high metastatic potential (L3.6sl), and a line closely resembling a primary tumor phenotype (Panc4.21) were examined [34]. All four lines express significant levels of non-phosphorylated ß-CATENIN, another reliable marker of active Wnt/ß-CATENIN signaling (data not shown). The requirement of Wnt/ß-CATENIN signaling for PDA cell proliferation and survival was assessed by ectopically expressing known pathway inhibitors. Co-transfection with the endogenous Wnt inhibitor, *Icat* [35] or a dominant negative form of *Lef-1* (*dn-Lef-1*), resulted in reduction of Top-Flash activity in the 4 PDA cell lines tested (Fig. 3A). In contrast, FOP-Flash activity was not affected by Icat or dn-Lef co-transfection (data not shown). Previous studies have shown effective reduction of Wnt/ß-CATENIN signaling in colon cancer cell lines upon treatment with siRNAs directed against *ß–CATENIN* [36]. Using a similar strategy, we observed a significant and dose dependant reduction in ß-CATENIN protein levels in pancreatic cancer cell lines transfected with anti-*ß-CATENIN* siRNA (Fig. 3B). Thus, Wnt/ß-CATENIN signaling in PDA cells can be blocked by treatment with three distinct and well-established pathway inhibitors.

Wnt/ß-CATENIN signaling controls cell proliferation and survival in tissues other than pancreas. To test whether inhibition of Wnt signaling is sufficient to block cell proliferation in pancreatic adenocarcinoma cells, we measured BrdU incorporation in cells transiently transfected with Wnt inhibitors *Icat* and *dn-Lef-1*. The presence of an *IRES* sequence driving the expression of *eGFP* in the *Icat*- and *dn-Lef-1*-constructs allowed sorting and comparison of GFP/target gene negative control and GFP/target gene positive cells by flow cytometry. Transfection of the four pancreatic cancer cell lines with either *Icat* or *dn-Lef-1* led to a significant decrease in cell proliferation and a marked increase in apoptosis (Fig. 3C, E). Similar results were obtained when ß-CATENIN function was specifically blocked with anti-*ß-CATENIN* siRNA [36] (Fig. 3D, F and Figure S3). Notably, we found that the reduction in cell proliferation and the increase in apoptosis upon ß-CATENIN knockdown was dose-dependent. The significant inhibition of cell proliferation and induction of apoptosis via inhibition of Wnt signaling through three different mechanisms strongly supports a role for Wnt signaling in PDA growth and survival.

**Figure 4:**
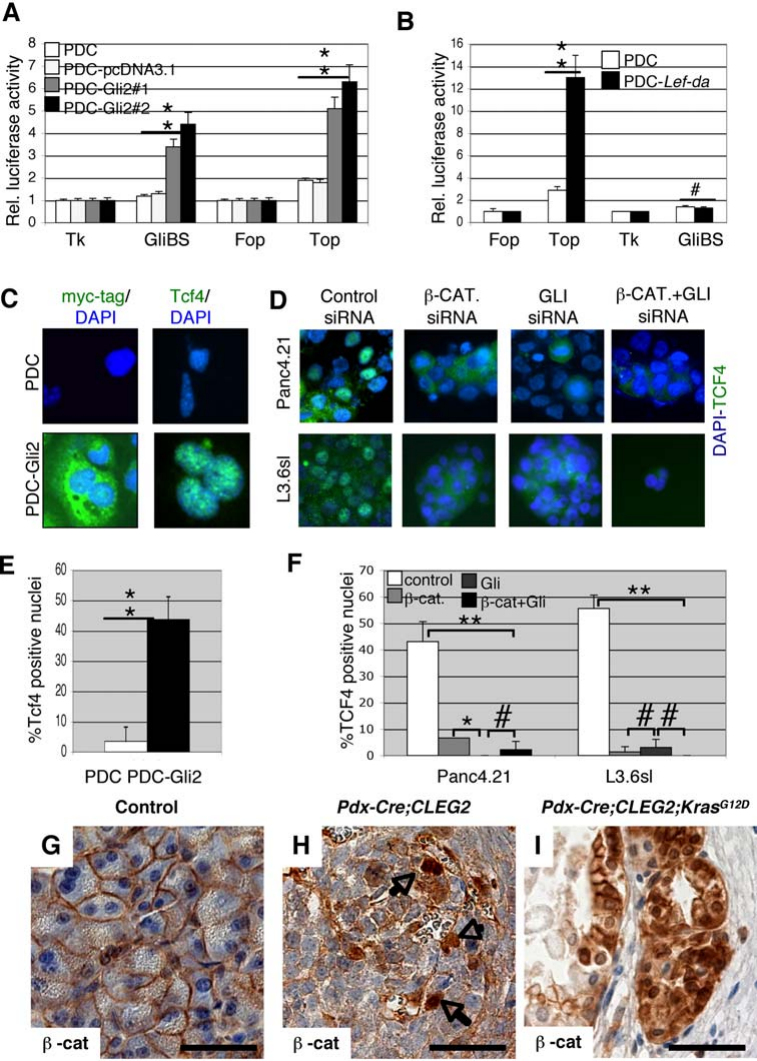
Hedgehog signaling regulates Wnt activity in untransformed duct cells. A. Activity of the Wnt and Hedgehog pathways in control PDCs and stable clones transfected with dominant-active forms of *GLI2* (PDC-*GLI2*). Activation of the Hedgehog pathway in two PDC-*GLI2* clones (#1, #2) results in significant increase in Wnt signaling activity. B. Activity of the Wnt and Hedgehog pathways in control PDCs and PDCs stably transfected with a dominant active form of *Lef1* (PDC-*Lef–da*). Activation of the Wnt pathway in the PDC-*Lef–da* cells does not affect the level of Hedgehog signaling. Error bars are shown as St. Dev. P-values, #, not significant; **p<0.01. C. PDC cells were stably transfected with a myc-tagged version of the constitutive active form of *GLI2* (GLI2-myc fusion protein). Immunostaining using an anti-myc antibody confirms GLI2 expression in transfected cells. Untransfected PDC cells are shown as negative control. Immunostaining for Tcf4 is not detectable in wt PDC cells, but it is strongly upregulated in PDC-*GLI2* cells. D. Immunohistochemistry analysis of Panc4.21 and L3.6sl pancreatic cancer cell lines shows that inhibition of the Wnt signaling pathway using anti-β-*CATENIN* siRNA (100nM) results in a strong downregulation of TCF4 expression compared to control cells (transfected with an unrelated siRNA). Similarly, inhibition of the Hedgehog pathway using an anti-*GLI1/2* siRNA (100 nM) results in TCF4 downregulation; the same effect is observed in cells treated with a combination of anti-β-CATENIN and anti-*GLI*1/2 siRNA (each 100nM). E. Quantification of Tcf4 expression in PDC (white) and PDC-GLI2 (black) cells. At least 100 cell nuclei were scored for Tcf4 expression and the percentage of positive nuclei is shown in the histogram. F. Quantification of TCF4 positive nuclei following siRNA treatment in human PDA cell lines. At least 100 nuclei were scored for each category: control siRNA (white), anti-β-CATENIN siRNA (light gray), anti-GLI siRNA (dark gray) and a combination of both siRNAs (black). Error bars are shown as St. Dev.; p-values #, not significant; *p<0.05; **, p<0.01. G. In wild-type mouse pancreas tissue, β-catenin is only localized at the cell membrane. H. Presence of nuclear β-catenin in a fraction of the tumors cells in an undifferentiated pancreatic tumor of a *Pdx-Cre;CLEG2* mouse. I. Cytoplasmic and nuclear β-catenin staining in the PanIN lesions of a *Pdx-Cre;CLEG2;Kras^G12D^* pancreas. The black bar represents 35 µm in G-I.

Previous reports have demonstrated an important role for ß-CATENIN in cell adhesion as part of a protein complex that includes E-cadherin. Therefore, disruption of this protein aggregate through ectopic expression of *Icat* or treatment with anti-ß-CATENIN siRNAs could affect cell viability by perturbing critical functions of E-cadherin rather than via decreased Wnt signaling. The E-CADHERIN expression pattern in the cell lines was unaffected by *ß-CATENIN*- siRNA knockdown (data not shown). In addition, a similar reduction in cell proliferation and increase in apoptosis was seen with *dn-Lef-1* transfection, that specifically blocks ß-CATENIN mediated transcription (Fig. 3C, E). These results suggest that inhibition of Wnt/ß-CATENIN transcriptional activity, rather than alteration in ß-CATENIN/E-CADHERIN interaction, is critical for the observed effects on cell proliferation and apoptosis.

### Hedgehog signaling regulates Wnt signaling in untransformed pancreatic ductal epithelial cells and in pancreatic cancer cells

Canonical Wnt/ß–catenin signaling is absent from normal mature pancreata [11] (Figure 1A, E). Our data and previously published reports show induction of ß-catenin signaling during PanIN formation, raising the question of the mechanism by which Wnt/ß-catenin signaling is induced. Hedgehog signaling, another embryonic signaling pathway, has previously been implicated in the initiation and growth of pancreatic adenocarcinoma [6], [7] as well as regulation of Wnt activity during mammalian organ development [37], [38]. Pancreatic ductal epithelial cells may constitute the putative cell of origin for PDA [39]. A previously described protocol allows the isolation of pancreatic duct cells (PDCs) from mature murine pancreas [40]. We used these cells to investigate the effect of Hedgehog signaling on Wnt activity in non-transformed epithelial cells. Ectopic activation of Hedgehog activity in PDCs was obtained either through expression of a dominant-active version of *GLI2* [41] or a dominant-active form of *Smoothened* (*SmoA1*). Expression of the transgenes was confirmed by immunofluorescence (Fig. 4C and data not shown). As expected, the activity of the Hedgehog pathway was significantly up-regulated in PDC-SmoA1 and PDC-GLI2 compared to PDCs, as measured by luciferase activity of a Hedgehog reporter construct, Gli-BS (Fig 4A and data not shown). Interestingly, the activity of the Wnt reporter TOP-Flash was also significantly increased (Fig 4A), thus showing that activation of Hedgehog signaling upregulates Wnt activity in pancreatic duct cells. These findings support the notion that the induction of Wnt activity in human PDA can occur in the absence of signature mutation in APC or ß-CATENIN that are commonly found in other cancers.

To test whether Wnt signaling regulates Hedgehog activity, PDCs were transfected with an expression vector coding for a dominant-active form of *Lef1*, (*Lef1-da*) followed by an *IRES-eGFP* sequence. Target gene expression in pools of transfected cells was verified by Western blot and by flow cytometry using eGFP as a marker (data not shown). Top–Flash reporter assays confirmed an approximately 4–fold increase in Wnt activity in PDC*–Lef1–da* cells compared to PDCs. However, ectopic activation of Wnt signaling did not induce luciferase activity of the Hedgehog responsive Gli-BS reporter (Fig. 4B).

### Hedgehog signaling regulates expression of TCF4 in pancreatic duct and cancer cells

While a number of studies have demonstrated that Hedgehog and Wnt pathways regulate each other's activity, the molecular mechanisms underlying this regulation remain unresolved. Activation of the canonical Wnt pathway requires the physical interaction of nuclear ß-CATENIN and TCF/LEF transcription factors. β−CATENIN and factors of the TCF/LEF family of DNA-binding molecules are the downstream effectors of the Wnt signaling cascade. TCF4 is expressed in the gastrointestinal tract and in tumors of gastrointestinal origin. Formation of the β−CATENIN/TCF4 complex is regulated at different levels, such as accumulation and localization of β−CATENIN [42], interaction between the two factors, and ability of TCF4 to bind DNA [43]. *TCF4* mRNA is expressed at high levels in all pancreatic adenocarcinoma cell lines analyzed (Fig. 2A). Little Tcf4 protein was detected in a small proportion of pancreatic duct cells, however, GLI2-mediated activation of Hedgehog signaling induced robust Tcf4 expression in pancreatic duct cells (Fig. 4C, E). To test whether Hedgehog signaling controls TCF4 expression in pancreatic cancer cells, we treated Panc4.21 and L3.6sl cells with siRNAs directed against *ß-CATENIN* as well as *GLI1/GLI2*. Interestingly, inhibition of either Hedgehog or Wnt signaling resulted in loss of nuclear TCF4 expression (Fig. 4D, F). In addition, treatment of the pancreatic cancer cells with the Hedgehog inhibitor cyclopamine resulted in a significant down-regulation of TCF4 protein and Top-Flash activity in the CFPAC, Panc4.21 and L3.6 cell lines (Fig. S4). The observation that TCF4 levels and Top-Flash activity were not affected in the cyclopamine insensitive cell line BxPC3 cells further support the notion that TCF4 expression is regulated in response to Hedgehog signaling. Thus, Hedgehog signaling regulates the Wnt pathway at least in part by affecting the concentration of nuclear TCF4 in pancreatic cancer cells.

To test whether Hedgehog activation is sufficient to induce Wnt/ß-catenin signaling *in vivo*, we analyzed transgenic mice marked by ectopic expression of an activated version of GLI2 in pancreatic epithelium (*Pdx-Cre;CLEG2*). We have previously reported that forced activation of Hedgehog signaling in *Pdx-Cre;CLEG2* mice results in the formation of undifferentiated tumors [44]. Immunohistochemical analysis revealed that tumor cells are marked by cytoplasmic and nuclear ß-catenin expression (Fig. 4H). Thus, epithelial-specific activation of Hedgehog signaling is sufficient to induce Wnt/ß-catenin signaling in pancreatic cells. Furthermore, nuclear ß-catenin is observed in PanIN as well as undifferentiated tumors that form in mice in which both Kras and Hedgehog signaling are deregulated (*Pdx-Cre;CLEG2;Kras^G12D^*, Fig. 4I). Summarily, our data from human, transgenic mouse and cell culture experiments suggest that Wnt/ß-catenin signaling is commonly activated in preneoplastic lesions and PDA and that the activation of the pathway is regulated at least in part by increased Hh signaling.

Notably, both Hedgehog and Wnt signaling appear to be important for the survival of pancreatic adenocarcinoma cells. This raises the question whether the cooperation between these pathways is restricted to the pancreas or whether similar mechanisms can also be found in other tumors. With the exception of the colon where conflicting results regarding the relationship between Hedgehog and Wnt signaling have been reported [45], [46], activation rather than inhibition of both pathways is typically correlated with tumor formation [47]. Hedgehog and Wnt activity have been noted in a number of different tumors and future studies will analyze whether combined antagonist treatments could present novel therapeutic options for these tumors. This is of particular importance as novel, more efficient and more specific inhibitors of both pathways are currently being developed [48], [49].

## Materials and Methods

### Mouse strains

*Pdx1-Cre^early^* mice ([10], [50]) were intercrossed with *CLEG2* mice (**A. Ermilov et al., in preparation ;** [44]) and with *LSL-Kras^G12D^* mice (a gift from David Tuveson referred to as Kras^G12D^ throughout the text) ([31]) to generate either double or triple mutants: *Pdx-Cre^early^;Kras^G12D^ , Pdx-Cre^early^;CLEG2* and *Pdx-Cre^early^;CLEG2;Kras^G12D^*. *Pdx-Cre^early^;Kras^G12D^* mice were crossed with the *Top-gal* Wnt reporter mice ([32]). All studies were conducted in compliance with University of California IACUC guidelines.

### Immunohistochemistry and Immunofluorescence

Histological analysis of tissues was performed as described previously [44]. The following primary antibodies were used: mouse anti-ß-catenin (1∶200 dilution, Becton and Dickinson, NJ), mouse anti-Tcf4 (clone 6H5-3, 1∶100 dilution, Upstate, NY). For immunohistochemistry, we used a biotinylated anti-mouse antibody (Jackson immunoresearch, PA) at a 1∶300 dilution. 3–3′-Diaminobenzidine tetrahydrochloride was used as a chromogen. Bright-field images were acquired using a Zeiss Axio Imager D1 scope. Alternatively, we used a FITC anti-mouse secondary antibody (Jackson immunoresearch, PA), and the slides were mounted using Vectashlield mounting medium vith DAPI (Vector laboratories, CA). Immunofluorescent images were acquired using an *Axioscop 2 plus* microscope (Zeiss, Germany).

### X-gal Staining

Mice were perfused with ice-cold fixative [0.25% glutaraldehyde in phosphate-buffered saline (PBS)]. Pancreas samples were fixed for 90 minutes at 4°C. Tissues were then washed with PBS containing 2 mM MgCl_2_ for 1 hour and stained with LacZ solution (1 mg/ml X-gal, 5 mM potassium ferricyanide, 5 mM potassium ferrocyanide, 2 mM MgCl2, 0.02%(v/v) NP40, and 0.01%(w/v) sodium-deoxycholate in PBS) at room temperature for 24 hours. After staining, tissues were post-fixed with buffered formalin, embedded in paraffin, sectioned, and counterstained with nuclear fast red.

### Cell culture and transfection

Tumor cell lines and cell culture protocols were previously described [7]. PDC cells growth conditions were previously published ([40] [51]). Cells were transfected using Effectene reagent (QIAGEN, Germany) according to the manufacturer's instructions. For transient transfections, the cells were harvested 24 to 48 hrs after transfection, and the luciferase and renilla acivity were assayed using the dual luciferase kit (Promega, WI). Stable transfectant cells were generated by selecting with G418 at a concentration of 300 µg/ml. Single clones were isolated and amplified.

### RT-PCR

RNA from pancreatic cancer cells and PDCs was prepared using TRIzol reagent (Invitrogen, CA). 2 µg of total RNA were treated with DNAse RQ1 (Promega) prior to cDNA synthesis (using random hexamers and Superscript II reverse transcriptase, (Invitrogen, CA). The oligonucleotide sequences are described in the supplemental information (Methods S1).

### Anti-sense RNAs

Anti-β-catenin and anti-GLI1 siRNA sequences were previously described ([36] [52]). The Gli2 anti-sense siRNA sequence was: 5′-ACACCAACCAGAACAAGCAdTdT-3′. The siRNAs were synthesized by Dharmacon, Inc. (CO). The siRNAs were transfected using oligofectamine reagent (Invitrogen, CA) according to the manifacturer's instructions.

### BrdU incorporation assay

BrdU incorporation assays were performing using the BD Biosciences BrdU flow kit according to the manufacturer's instructions.

Additional methods are provided in Methods S1.

## Supporting Information

Figure S1 Immunostaining of β-CATENIN in human normal and cancer tissues. A. In control human pancreas β-CATENIN is localized at the cell membrane in both acinar and duct cells. B, C, D. In three different human PDA samples β-CATENIN is localized predominantly in the cytoplasm (D) and nucleus (B,C).
(9.42 MB TIF)

Figure S2 Quantitative PCR for Wnt target genes (axin2, Lef1, Mmp-7, cyclinD1, Dkk2 and Dkk3) in pancreatic samples isolated from three wild type control and three Pdx-Cre;KrasG12D mice. All mice analyzed were 6 months old. Data are presented compared to expression of the household gene GUS.
(0.36 MB TIF)

Figure S3 Inhibition of Wnt signaling induces apoptosis in pancreatic cancer cell lines. A. Relative apoptosis is measured by FACS using an anti-cleaved caspase 3 antibody in cells transfected with a control siRNA (control, white bars) or β-catenin siRNA (black bars). B. Immunostaining for cleaved caspase 3 in cells transfected with a control siRNA or β-catenin siRNA.
(1.78 MB TIF)

Figure S4 The Hedgehog signaling pathway acts upstream of the Wnt signaling pathway in pancreatic cancer cells. A. Activity of the Top-Flash vector in pancreatic adenocarcinoma cells (black bars) is inhibited in response to cyclopamine (gray bars), an inhibitor of the Hedgehog signaling pathway. The first P-values indicate statistical significance in Top-Flash activation (black bars) in comparison to Fop-Flash activity (white bars). The second P-values show significance of reduction in Top-Flash activity upon cyclopamine treatment (gray vs black bars). B. Immunostaining of pancreatic cancer cells grown in control conditions or treated with cyclopamine for 24 hrs. With the exception of BxPC# cells, cyclopamine treatment strongly inhibits TCF4 expression in the cell lines analyzed. Green: anti-TCF4 antibody; blue: DAPI. C. Quantification of the percentage of TCF4 positive nuclei in control cells (white bars) and in cyclopamine-treated cells (black bars). P-values are shown in comparison to the control cells. Error bars are shown as St. Dev.; p-values #, not significant; *p<0.05; **, p<0.01.
(1.71 MB TIF)

Table S1 Upregulation of Wnt receptors, Wnt ligands and β-CATENIN itself as well as downregulation of some inhibitors of Wnt signaling (IDAX and ICAT), together with genes whose transcription is mediated by β-CATENIN, suggests activation of Wnt signaling in PDA.
(0.18 MB DOC)

Methods S1 (0.05 MB DOC)

## References

[1] Hezel AF, Kimmelman AC, Stanger BZ, Bardeesy N, Depinho RA (2006). Genetics and biology of pancreatic ductal adenocarcinoma.. Genes Dev.

[2] Hruban RH, Takaori K, Klimstra DS, Adsay NV, Albores-Saavedra J (2004). An illustrated consensus on the classification of pancreatic intraepithelial neoplasia and intraductal papillary mucinous neoplasms.. Am J Surg Pathol.

[3] Biankin AV, Kench JG, Dijkman FP, Biankin SA, Henshall SM (2003). Molecular pathogenesis of precursor lesions of pancreatic ductal adenocarcinoma.. Pathology.

[4] Biankin AV, Kench JG, Morey AL, Lee CS, Biankin SA (2001). Overexpression of p21(WAF1/CIP1) is an early event in the development of pancreatic intraepithelial neoplasia.. Cancer Res.

[5] Miyamoto Y, Maitra A, Ghosh B, Zechner U, Argani P (2003). Notch mediates TGF alpha-induced changes in epithelial differentiation during pancreatic tumorigenesis.. Cancer Cell.

[6] Berman DM, Karhadkar SS, Maitra A, Montes de Oca R, Gerstenblith MR (2003). Widespread requirement for Hedgehog ligand stimulation in growth of digestive tract tumours.. Nature.

[7] Thayer SP, di Magliano MP, Heiser PW, Nielsen CM, Roberts DJ (2003). Hedgehog is an early and late mediator of pancreatic cancer tumorigenesis.. Nature.

[8] Pasca di Magliano M, Hebrok M (2004). Hedgehog signaling in cancer formation and maintenance.. Nature Reviews Cancer.

[9] Hebrok M (2003). Hedgehog signaling in pancreas development.. Mech Dev.

[10] Heiser PW, Lau J, Taketo MM, Herrera PL, Hebrok M (2006). Stabilization of beta-catenin impacts pancreas growth.. Development.

[11] Murtaugh LC, Law AC, Dor Y, Melton DA (2005). {beta}-Catenin is essential for pancreatic acinar but not islet development.. Development.

[12] Dessimoz J, Bonnard C, Huelsken J, Grapin-Botton A (2005). Pancreas-Specific Deletion of beta-Catenin Reveals Wnt-Dependent and Wnt-Independent Functions during Development.. Curr Biol.

[13] Papadopoulou S, Edlund H (2005). Attenuated wnt signaling perturbs pancreatic growth but not pancreatic function.. Diabetes.

[14] Apelqvist A, Ahlgren U, Edlund H (1997). Sonic hedgehog directs specialised mesoderm differentiation in the intestine and pancreas.. Curr Biol.

[15] Kawahira H, Scheel DW, Smith SB, German MS, Hebrok M (2005). Hedgehog signaling regulates expansion of pancreatic epithelial cells.. Dev Biol.

[16] Heller RS, Dichmann DS, Jensen J, Miller C, Wong G (2002). Expression patterns of Wnts, Frizzleds, sFRPs, and misexpression in transgenic mice suggesting a role for Wnts in pancreas and foregut pattern formation.. Dev Dyn.

[17] Nusse R (2003). Wnts and Hedgehogs: lipid-modified proteins and similarities in signaling mechanisms at the cell surface.. Development.

[18] Gregorieff A, Clevers H (2005). Wnt signaling in the intestinal epithelium: from endoderm to cancer.. Genes Dev.

[19] Lustig B, Behrens J (2003). The Wnt signaling pathway and its role in tumor development.. J Cancer Res Clin Oncol.

[20] Bafico A, Liu G, Goldin L, Harris V, Aaronson SA (2004). An autocrine mechanism for constitutive Wnt pathway activation in human cancer cells.. Cancer Cell.

[21] Abraham SC, Klimstra DS, Wilentz RE, Yeo CJ, Conlon K (2002). Solid-pseudopapillary tumors of the pancreas are genetically distinct from pancreatic ductal adenocarcinomas and almost always harbor beta-catenin mutations.. Am J Pathol.

[22] Anderson CB, Neufeld KL, White RL (2002). Subcellular distribution of Wnt pathway proteins in normal and neoplastic colon.. Proc Natl Acad Sci U S A.

[23] Al-Aynati MM, Radulovich N, Riddell RH, Tsao MS (2004). Epithelial-cadherin and beta-catenin expression changes in pancreatic intraepithelial neoplasia.. Clin Cancer Res.

[24] Lowy AM, Fenoglio-Preiser C, Kim OJ, Kordich J, Gomez A (2003). Dysregulation of beta-catenin expression correlates with tumor differentiation in pancreatic duct adenocarcinoma.. Ann Surg Oncol.

[25] Nawroth R, van Zante A, Cervantes S, McManus M, Hebrok M (2007). Extracellular sulfatases, elements of the wnt signaling pathway, positively regulate growth and tumorigenicity of human pancreatic cancer cells.. PLoS ONE.

[26] Wells JM, Esni F, Boivin GP, Aronow BJ, Stuart W (2007). Wnt/beta-catenin signaling is required for development of the exocrine pancreas.. BMC Dev Biol.

[27] Rulifson IC, Karnik SK, Heiser PW, ten Berge D, Gu X (2007). Wnt signaling regulates pancreatic ß-cell proliferation.. Proc Natl Acad Sci U S A in press.

[28] Zeng G, Germinaro M, A. M, Monga NK, Bell A (2006). Aberrant Wnt/ß-Catenin Signaling in Pancreatic Adenocarcinoma.. Neoplasia.

[29] Klimstra DS, Longnecker DS (1994). K-ras mutations in pancreatic ductal proliferative lesions.. Am J Pathol.

[30] Rozenblum E, Schutte M, Goggins M, Hahn SA, Panzer S (1997). Tumor-suppressive pathways in pancreatic carcinoma.. Cancer Res.

[31] Hingorani SR, Petricoin EF, Maitra A, Rajapakse V, King C (2003). Preinvasive and invasive ductal pancreatic cancer and its early detection in the mouse.. Cancer Cell.

[32] DasGupta R, Fuchs E (1999). Multiple roles for activated LEF/TCF transcription complexes during hair follicle development and differentiation.. Development.

[33] van de Wetering M, Cavallo R, Dooijes D, van Beest M, van Es J (1997). Armadillo coactivates transcription driven by the product of the Drosophila segment polarity gene dTCF.. Cell.

[34] Jaffee EM, Schutte M, Gossett J, Morsberger LA, Adler AJ (1998). Development and characterization of a cytokine-secreting pancreatic adenocarcinoma vaccine from primary tumors for use in clinical trials.. Cancer J Sci Am.

[35] Tago K, Nakamura T, Nishita M, Hyodo J, Nagai S (2000). Inhibition of Wnt signaling by ICAT, a novel beta-catenin-interacting protein.. Genes Dev.

[36] Verma UN, Surabhi RM, Schmaltieg A, Becerra C, Gaynor RB (2003). Small interfering RNAs directed against beta-catenin inhibit the in vitro and in vivo growth of colon cancer cells.. Clin Cancer Res.

[37] Pinson KI, Brennan J, Monkley S, Avery BJ, Skarnes WC (2000). An LDL-receptor-related protein mediates Wnt signalling in mice.. Nature.

[38] Parr BA, McMahon AP (1995). Dorsalizing signal Wnt-7a required for normal polarity of D-V and A-P axes of mouse limb.. Nature.

[39] Hruban RH, Adsay NV, Albores-Saavedra J, Compton C, Garrett ES (2001). Pancreatic intraepithelial neoplasia: a new nomenclature and classification system for pancreatic duct lesions.. Am J Surg Pathol.

[40] Schreiber FS, Deramaudt TB, Brunner TB, Boretti MI, Gooch KJ (2004). Successful growth and characterization of mouse pancreatic ductal cells: functional properties of the Ki-RAS(G12V) oncogene.. Gastroenterology.

[41] Roessler E, Ermilov AN, Grange DK, Wang A, Grachtchouk M (2005). A previously unidentified amino terminal domain regulates transcriptional activity of wild-type and disease-associated human GLI2.. Hum Mol Genet.

[42] Hecht A, Kemler R (2000). Curbing the nuclear activities of beta-catenin. Control over Wnt target gene expression.. EMBO Rep.

[43] Ishitani T, Ninomiya-Tsuji J, Matsumoto K (2003). Regulation of lymphoid enhancer factor 1/T-cell factor by mitogen-activated protein kinase-related Nemo-like kinase-dependent phosphorylation in Wnt/beta-catenin signaling.. Mol Cell Biol.

[44] Pasca di Magliano M, Sekine S, Ermilov A, Ferris J, Dlugosz AA (2006). Hedgehog/Ras interactions regulate early stages of pancreatic cancer.. Genes Dev.

[45] van den Brink GR, Bleuming SA, Hardwick JC, Schepman BL, Offerhaus GJ (2004). Indian Hedgehog is an antagonist of Wnt signaling in colonic epithelial cell differentiation.. Nat Genet.

[46] Qualtrough D, Buda A, Gaffield W, Williams AC, Paraskeva C (2004). Hedgehog signalling in colorectal tumour cells: induction of apoptosis with cyclopamine treatment.. Int J Cancer.

[47] Taipale J, Beachy PA (2001). The Hedgehog and Wnt signalling pathways in cancer.. Nature.

[48] Frank-Kamenetsky M, Zhang XM, Bottega S, Guicherit O, Wichterle H (2002). Small-molecule modulators of Hedgehog signaling: identification and characterization of Smoothened agonists and antagonists.. J Biol.

[49] Lepourcelet M, Chen YN, France DS, Wang H, Crews P (2004). Small-molecule antagonists of the oncogenic Tcf/beta-catenin protein complex.. Cancer Cell.

[50] Gu G, Dubauskaite J, Melton DA (2002). Direct evidence for the pancreatic lineage: NGN3+ cells are islet progenitors and are distinct from duct progenitors.. Development.

[51] Deramaudt TB, Takaoka M, Upadhyay R, Bowser MJ, Porter J (2006). N-cadherin and keratinocyte growth factor receptor mediate the functional interplay between Ki-RASG12V and p53V143A in promoting pancreatic cell migration, invasion, and tissue architecture disruption.. Mol Cell Biol.

[52] Sanchez P, Hernandez AM, Stecca B, Kahler AJ, DeGueme AM (2004). Inhibition of prostate cancer proliferation by interference with SONIC HEDGEHOG-GLI1 signaling.. Proc Natl Acad Sci U S A.

