# Supplementary figures and images for: Common Activation of Canonical Wnt Signaling in Pancreatic Adenocarcinoma
Source: PLoS One. 2007 Nov 7;2(11):e1155. doi: 10.1371/journal.pone.0001155 (PMC2048934; doi:10.1371/journal.pone.0001155)

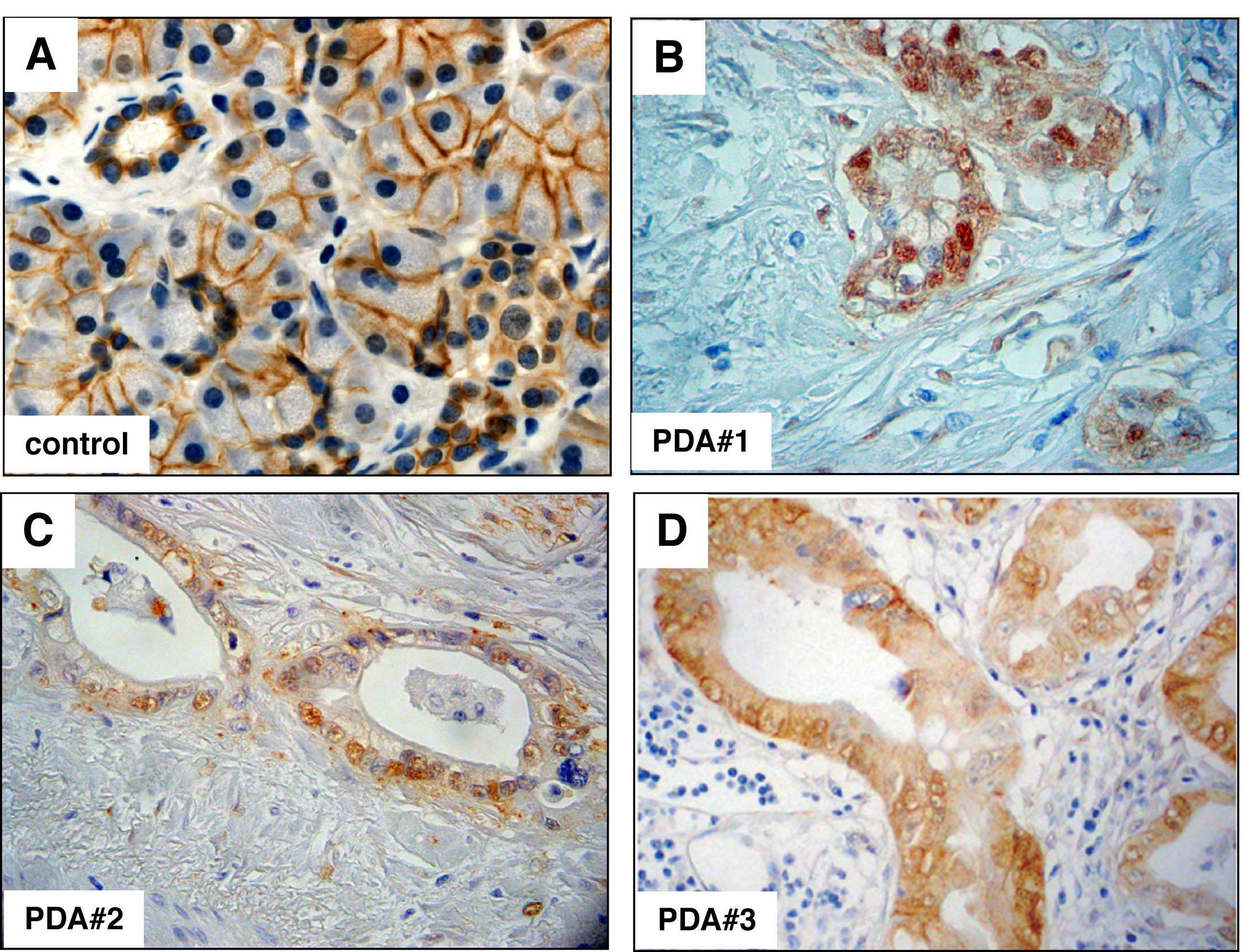

Supplement: Figure S1 — Immunostaining of β-CATENIN in human normal and cancer tissues. A. In control human pancreas β-CATENIN is localized at the cell membrane in both acinar and duct cells. B, C, D. In three different human PDA samples β-CATENIN is localized predominantly in the cytoplasm (D) and nucleus (B,C). (9.42 MB TIF) [file pone.0001155.s001.tif]

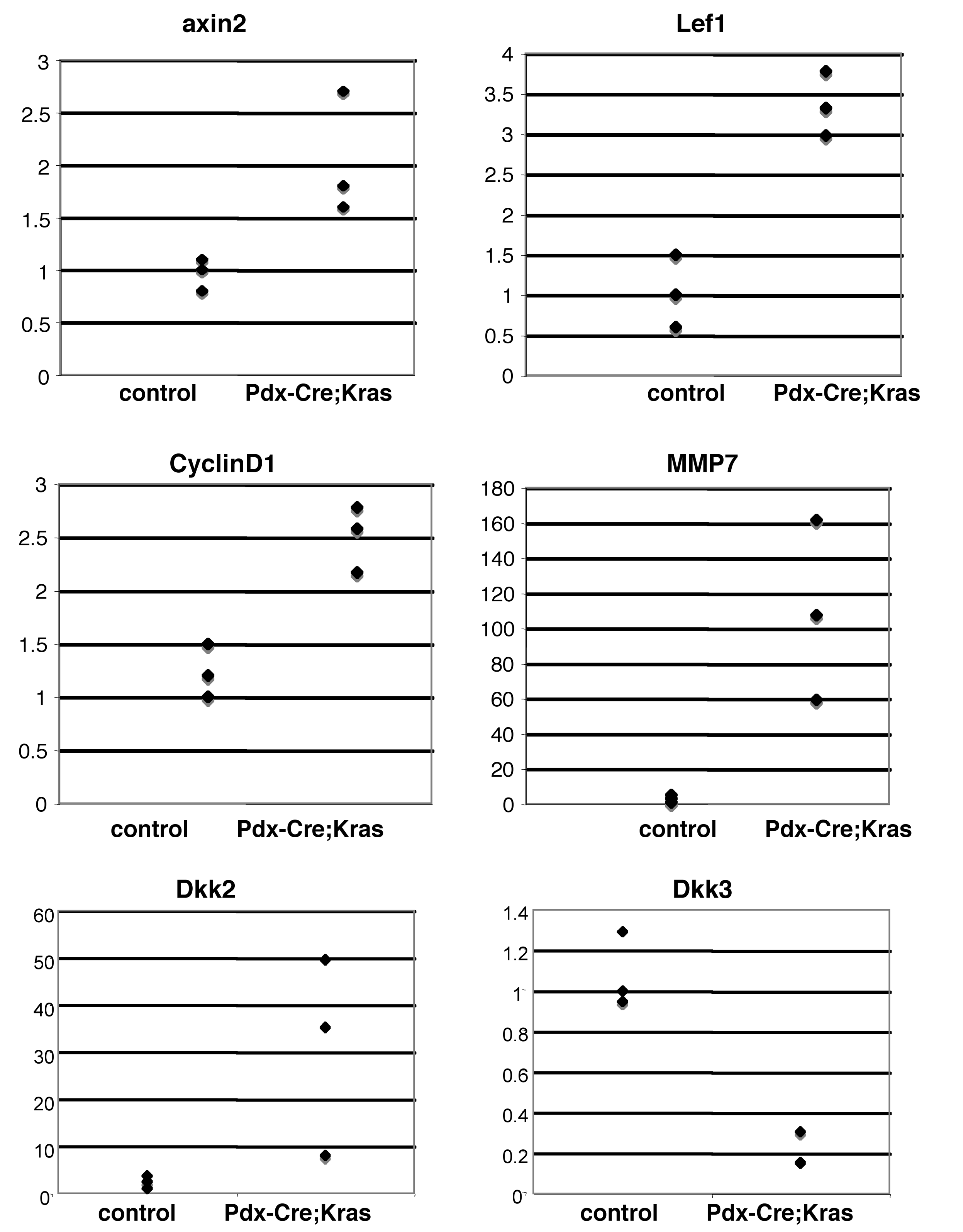

Supplement: Figure S2 — Quantitative PCR for Wnt target genes (axin2, Lef1, Mmp-7, cyclinD1, Dkk2 and Dkk3) in pancreatic samples isolated from three wild type control and three Pdx-Cre;KrasG12D mice. All mice analyzed were 6 months old. Data are presented compared to expression of the household gene GUS. (0.36 MB TIF) [file pone.0001155.s002.tif]

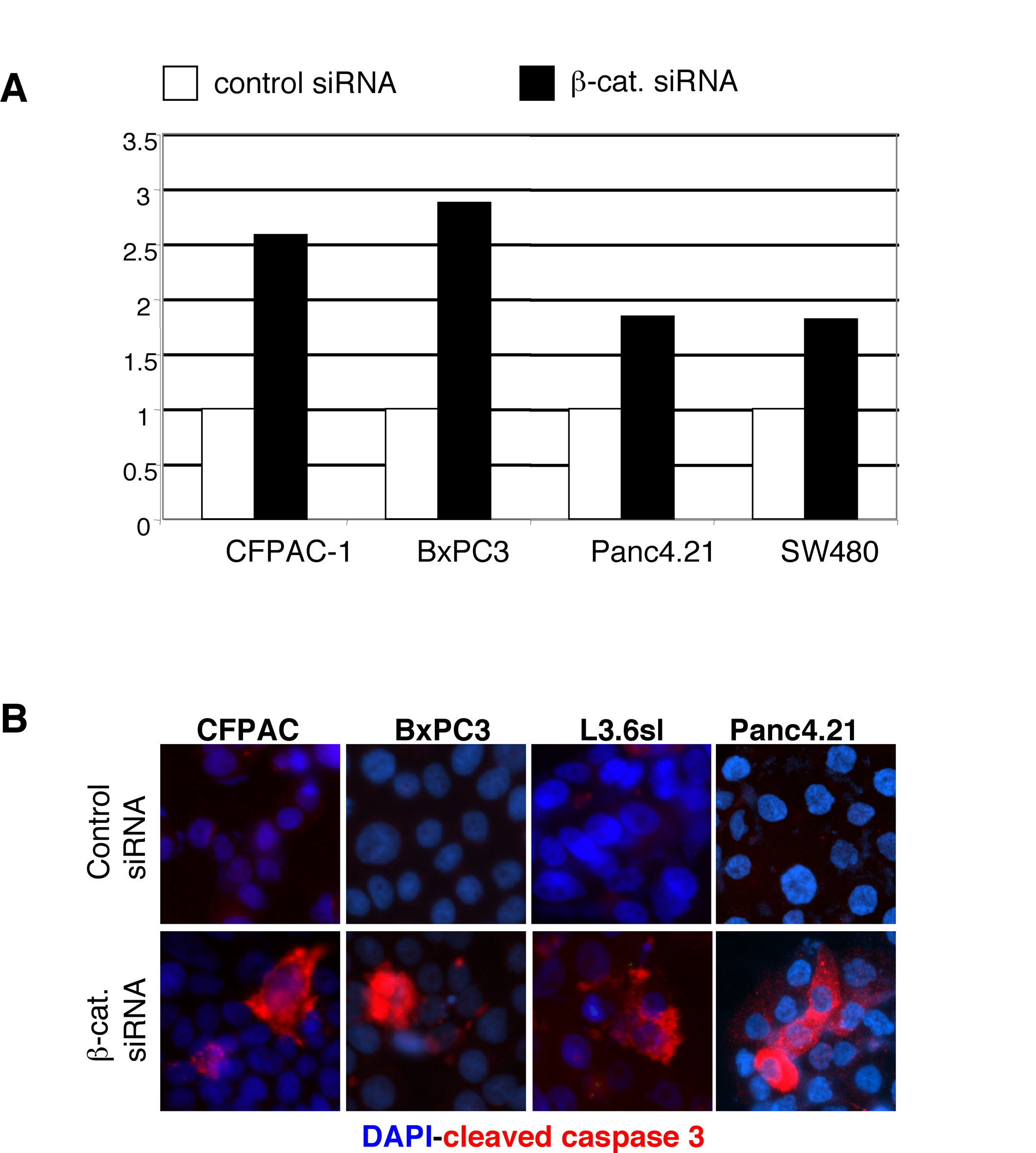

Supplement: Figure S3 — Inhibition of Wnt signaling induces apoptosis in pancreatic cancer cell lines. A. Relative apoptosis is measured by FACS using an anti-cleaved caspase 3 antibody in cells transfected with a control siRNA (control, white bars) or β-catenin siRNA (black bars). B. Immunostaining for cleaved caspase 3 in cells transfected with a control siRNA or β-catenin siRNA. (1.78 MB TIF) [file pone.0001155.s003.tif]

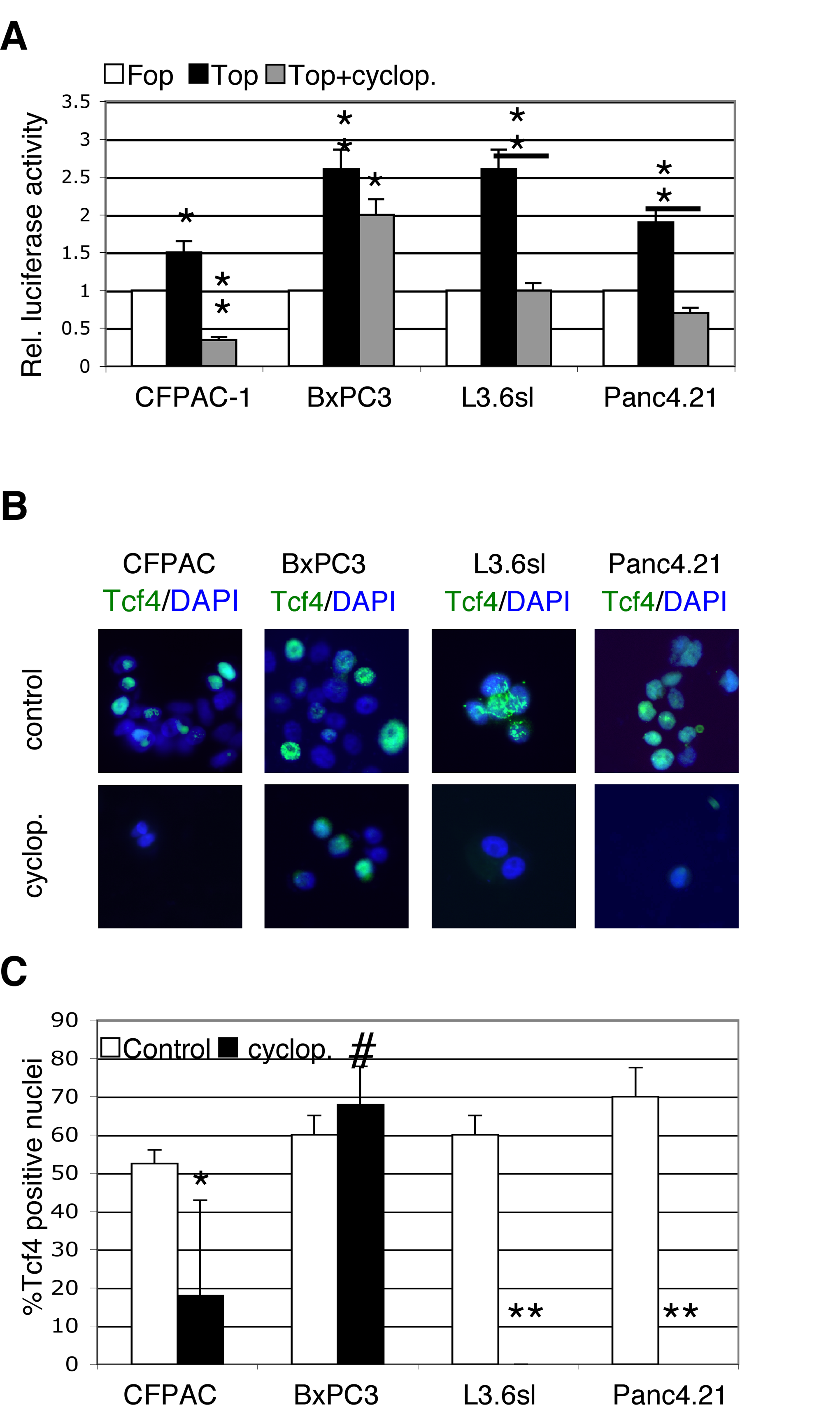

Supplement: Figure S4 — The Hedgehog signaling pathway acts upstream of the Wnt signaling pathway in pancreatic cancer cells. A. Activity of the Top-Flash vector in pancreatic adenocarcinoma cells (black bars) is inhibited in response to cyclopamine (gray bars), an inhibitor of the Hedgehog signaling pathway. The first P-values indicate statistical significance in Top-Flash activation (black bars) in comparison to Fop-Flash activity (white bars). The second P-values show significance of reduction in Top-Flash activity upon cyclopamine treatment (gray vs black bars). B. Immunostaining of pancreatic cancer cells grown in control conditions or treated with cyclopamine for 24 hrs. With the exception of BxPC# cells, cyclopamine treatment strongly inhibits TCF4 expression in the cell lines analyzed. Green: anti-TCF4 antibody; blue: DAPI. C. Quantification of the percentage of TCF4 positive nuclei in control cells (white bars) and in cyclopamine-treated cells (black bars). P-values are shown in comparison to the control cells. Error bars are shown as St. Dev.; p-values #, not significant; *p<0.05; **, p<0.01. (1.71 MB TIF) [file pone.0001155.s004.tif]
