# Supplementary material for: Common Activation of Canonical Wnt Signaling in Pancreatic Adenocarcinoma
Source: PLoS One. 2007 Nov 7;2(11):e1155. doi: 10.1371/journal.pone.0001155 (PMC2048934; doi:10.1371/journal.pone.0001155)
Supplement: Table S1 — Upregulation of Wnt receptors, Wnt ligands and β-CATENIN itself as well as downregulation of some inhibitors of Wnt signaling (IDAX and ICAT), together with genes whose transcription is mediated by β-CATENIN, suggests activation of Wnt signaling in PDA. (0.18 MB DOC) [file pone.0001155.s005.doc]

**Suppl. Table 1: Expression of transcripts in PDA compared to normal pancreas.**

| **Affymetrix Probe** | **Unigene Cluster** | **Gene** | **Fold Change** | **t test**  **P value** |
| --- | --- | --- | --- | --- |
| **204451_at** | **Hs.94234** | **frizzled (Drosophila) homolog 1 (FZD1)** | **1.7** | ***0.047*** |
| **210220_at** | **Hs.81217** | **frizzled (Drosophila) homolog 2 (FZD2)** | **4.2** | ***0.019*** |
| 219683_at | Hs.40735 | frizzled (Drosophila) homolog 3 (FZD3) | 1.1 | *0.777* |
| 218665_at | Hs.19545 | frizzled (Drosophila) homolog 4 (FZD4) | 0.7 | *0.166* |
| 206136_at | Hs.152251 | frizzled (Drosophila) homolog 5 (FZD5) | 1.3 | *0.569* |
| 203987_at | Hs.114218 | frizzled (Drosophila) homolog 6 (FZD6) | 1.3 | *0.207* |
| **203705_s_at** | **Hs.173859** | **frizzled (Drosophila) homolog 7 (FZD7)** | **2.1** | ***0.004*** |
| 216587_s_at |  | frizzled (Drosophila) homolog 8 (FZD8) | 0.9 | *0.701* |
| 207639_at | Hs.158335 | frizzled (Drosophila) homolog 9 (FZD9) | 2 | *0.101* |
| 219764_at | Hs.31664 | frizzled (Drosophila) homolog 10 (FZD10) | 0.9 | *0.829* |
| 209468_at | Hs.6347 | Lipoprotein Receptor Related Protein 5 (LRP5) | 1.2 | *0.682* |
| 34697_at | Hs.23672 | Lipoprotein Receptor Related Protein 6 (LRP6) | 1.3 | *0.507* |
| 208570_at | Hs.248164 | WNT-1 | 1.3 | *0.599* |
| **205648_at** | **Hs.89791** | **WNT-2** | **3.1** | ***0.005*** |
| 206458_s_at | Hs.258575 | WNT-2B | 0.7 | *0.314* |
| 221455_s_at | Hs.224667 | WNT-3 | 2.1 | *0.199* |
| 208606_s_at | Hs.302428 | WNT-4 | 3.1 | *0.138* |
| **213425_at** | **Hs.152213** | **WNT-5A** | **2.4** | ***0.023*** |
| **221029_s_at** | **Hs.306051** | **WNT-5B** | **2.6** | ***0.007*** |
| 222086_s_at | Hs.29764 | WNT-6 | 3.9 | *0.332* |
| 217681_at | Hs.65905 | WNT-7A | 1.3 | *0.597* |
| 207612_at | Hs.137595 | WNT-8B | 2.5 | *0.303* |
| 206213_at | Hs.91985 | WNT-10B | 0.8 | *0.493* |
| 206737_at | Hs.108219 | WNT-11 | 2.6 | *0.128* |
| 221113_s_at | Hs.272375 | WNT-16 | 1.4 | *0.406* |
| 204602_at | Hs.40499 | dickkopf (Xenopus laevis) homolog 1 (DKK1) | 4.1 | *0.057* |
| 219908_at | Hs.211869 | dickkopf (Xenopus laevis) homolog 2 (DKK2) | 3.1 | *0.069* |
| **202196_s_at** | **Hs.4909** | **dickkopf (Xenopus laevis) homolog 3 (DKK3)** | **4.3** | ***<0.0001*** |
| 206619_at | Hs.159311 | dickkopf (Xenopus laevis) homolog 4 (DKK4) | 1.8 | *0.321* |
| 202035_s_at | Hs.7306 | secreted frizzled-related protein 1 (sFRP1) | 0.6 | *0.149* |
| **223122_s_at** | **Hs.31386** | **secreted frizzled-related protein 2 (sFRP2)** | **6.5** | ***0.007*** |
| **203697_at** | **Hs.153684** | **secreted frizzled-related protein 3 (sFRP3)** | **2.9** | ***0.004*** |
| **204052_s_at** | **Hs.105700** | **secreted frizzled-related protein 4 (sFRP4)** | **6.7** | ***0.042*** |
| **207468_s_at** | **Hs.279565** | **secreted frizzled-related protein 5 (sFRP5)** | **0.3** | ***<0.0001*** |
| 214633_at | Hs.348820 | SOX 3 | 0.8 | *0.738* |
| **204712_at** | **Hs.284122** | ***Wnt* Inhibitory Factor 1 (WIF-1)** | **0.4** | ***0.048*** |
| 201533_at | Hs.171271 | b-CATENIN (CTNNB1) | 2 | *0.154* |
| 202332_at | Hs.79658 | casein kinase 1, epsilon (CSNK1E) | 0.7 | *0.184* |
| **203230_at** | **Hs.74375** | **dishevelled 1 (homologous to Drosophila dsh) (DVL1)** | **0.6** | ***<0.0001*** |
| 218759_at | Hs.118640 | dishevelled 2 (homologous to Drosophila dsh) (DVL2) | 0.8 | *0.597* |
| 201908_at | Hs.174044 | dishevelled 3 (homologous to Drosophila dsh) (DVL3) | 1.3 | *0.059* |
| **219889_at** | **Hs.126057** | **GSK-3b binding protein FRAT1** | **0.6** | ***0.013*** |
| 209864_at | Hs.140720 | GSK-3b binding protein FRAT2 | 1 | *0.948* |
| 203338_at | Hs.173328 | protein phosphatase 2A, epsilon | 1.1 | *0.594* |
| 214083_at | Hs.171734 | protein phosphatase 2A, gamma | 2 | *0.098* |
| **210511_at** | **Hs.73888** | **transcription factor 1 (TCF1)** | **0.3** | ***0.032*** |
| **210776_x_at** | **Hs.101047** | **transcription factor 3 (TCF3)** | **3.1** | ***0.005*** |
| **203753_at** | **Hs.326198** | **transcription factor 4 (TCF4)** | **3.9** | ***0.03*** |
| **221558_s_at** | **Hs.44865** | **lymphoid enhancer factor 1 (LEF1)** | **9.3** | ***0.001*** |
| 203526_s_at | Hs.75081 | Adenomatos Polyposis Coli (APC) | 1 | *0.916* |
| 212849_at | Hs.184434 | Axin | 1.4 | *0.185* |
| 204901_at | Hs.226434 | beta-transducin repeat containing protein (b-trcp) | 1.4 | *0.223* |
| **220277_at** | **Hs.118569** | **IDAX (inhibition of the Dvl and Axin complex)** | **0.3** | ***0.032*** |
| **209945_s_at** | **Hs.78802** | **glycogen synthase kinase 3 beta (GSK-3b)** | **2.4** | ***0.01*** |
| **203081_at** | **Hs.99816** | **ICAT** | **0.5** | ***0.011*** |
| **207974_s_at** | **Hs.227950** | **S-phase kinase-associated protein 1A (p19A) (SKP1A)** | **1.6** | ***0.045*** |
| 204489_s_at | Hs.169610 | CD44 antigen (CD44) | 1.7 | *0.068* |
| 218182_s_at | Hs.7327 | claudin 1 | 1.2 | *0.609* |
| **204748_at** | **Hs.196384** | **Cyclo-oxygenase 2 (COX-2)** | **4** | ***0.007*** |
| 214019_at | Hs.82932 | cyclin D1 | 2.4 | *0.305* |
| 200952_s_at | Hs.75586 | cyclin D2 | 2.4 | *0.242* |
| 201700_at | Hs.83173 | cyclin D3 | 1.1 | *0.653* |
| **210495_x_at** | **Hs.287820** | **fibronectin 1** | **12** | ***<0.0001*** |
| **204948_s_at** | **Hs.9914** | **follistatin** | **0.5** | ***0.009*** |
| 201566_x_at | Hs.180919 | Id-2 | 1 | *0.896* |
| **204259_at** | **Hs.2256** | **matrix metalloproteinase 7 (MMP7)** | **4.2** | ***0.014*** |
| 220541_at | Hs.204732 | matrix metalloproteinase 26 (MMP26) | 0.7 | *0.221* |
| 208044_s_at | Hs.106415 | peroxisome proliferative activated receptor,delta | 1.1 | *0.791* |
| 204188_s_at | Hs.1497 | retinoic acid receptor gamma (RARg) | 2.2 | *0.059* |
| **211924_s_at** | **Hs.179657** | **Urokinase Plasminogen Activator Receptor (uPA-R)** | **13.5** | ***0.037*** |
| **202431_s_at** | **Hs.79070** | **c-myc** | **0.3** | ***0.015*** |
| **206796_at** | **Hs.194680** | **WISP1** | **3.3** | ***0.009*** |
| 205792_at | Hs.194679 | WISP2 | 0.7 | *0.561* |
| **210861_s_at** | **Hs.194678** | **WISP 3 (LIBC)** | **4.6** | ***0.037*** |
| 206923_at | Hs.169449 | protein kinase C, alpha (PRKCA), | 1.9 | *0.103* |
| 206270_at | Hs.2890 | protein kinase C, gamma (PRKCG) | 1.3 | *0.649* |
| 202545_at | Hs.155342 | protein kinase C, delta (PRKCD) | 0.7 | *0.194* |
| **206248_at** | **Hs.211592** | **protein kinase C, epsilon (PRKCE)** | **0.4** | ***0.013*** |
| 206099_at | Hs.315366 | protein kinase C, eta (PRKCH) | 0.8 | *0.461* |
| 209677_at | Hs.1904 | protein kinase C iota isoform (PRKCI) | 1.3 | *0.42* |
| **205880_at** | **Hs.2891** | **protein kinase C, mu (PRKCM)** | **2.2** | ***0.046*** |
| 210039_s_at | Hs.211593 | protein kinase C, theta | 0.6 | *0.054* |
| **202178_at** | **Hs.78793** | **protein kinase C, zeta (PRKCZ),** | **0.6** | ***0.05*** |
| **208640_at** | **Hs.173737** | **rho family, small GTP binding protein Rac-1** | **2** | ***0.005*** |

Upregulation of *Wnt* receptors, *Wnt* ligands and ß-CATENIN itself as well as downregulation of some inhibitors of *Wnt* signaling (IDAX and ICAT), together with genes whose transcription is mediated by ß-CATENIN, suggests activation of *Wnt* signaling in PDA.
