# Supplementary material for: Common Activation of Canonical Wnt Signaling in Pancreatic Adenocarcinoma
Source: PLoS One. 2007 Nov 7;2(11):e1155. doi: 10.1371/journal.pone.0001155 (PMC2048934; doi:10.1371/journal.pone.0001155)
Supplement: Methods S1 — (0.05 MB DOC) [file pone.0001155.s006.doc]

## Supplementary Materials and Methods

#### **Expression Profiling**

#### ***RNA preparation and transcript profiling***

Human tissue acquisition and global analysis of gene expression experiments were performed as previously described ^1^. Briefly, ethical approval was obtained from five teaching hospitals (The Princess Alexandra Hospital, Brisbane, Australia, Westmead Hospital, Concord Hospital, Royal Prince Alfred Hospital and St. Vincent’s Hospital Campus in Sydney, Australia) for the acquisition of fresh and archival tissue and recording of clinicopathological data. Multiple samples of pancreatic tissue of approximately 500 mg were excised intraoperatively from 12 patients, undergoing pancreatic resection for PC, immediately snap frozen in liquid nitrogen and stored at minus 80^o^C, prior to RNA extraction. Total RNA was isolated from 12 PC specimens and 6 macroscopically and microscopically normal appearing pancreas from the same patients (matched). Biotinylated cRNA for Affymetrix Genechip® hybridization was prepared through a single round of reverse transcription with Superscript II (Life Technologies, Rockville, MD) followed by second strand synthesis to create double stranded cDNA. After purification the cDNA was transcribed and labelled using a T7 polymerase (Enzo Technologies, New York, NY) and purified. Hybridization cocktails were prepared as per the Affymetrix protocol (Affymetrix, Santa Clara, CA) and quality assured on Affymetrix Test3 arrays, prior to hybridization to HG-U133A and B oligonucleotide microarrays.

**Tissue library**

We identified a cohort of 136 patients diagnosed with pancreatic adenocarcinoma who underwent pancreatic resection or biopsy from Westmead Hospital, Concord Hospital, The Royal Prince Alfred Hospital and The St Vincent’s Hospital Campus in Sydney, Australia. This cohort represents a subset of a previously described group of 348 patients ^1-3^. Archival, formalin-fixed, paraffin-embedded tissue from pancreata that were resected or biopsied between January 1972 and November 2001 was used to construct PC tissue arrays which contained up to 55 x 1.6 mm cores per slide with 1 to 3 cores per patient sample.

**RT-PCR Oligonucleotides**

WNT11

sense 5’-CAC TGA ACC AGA CGC AAC AC-3’

antisense 5’-ATA CAC GAA GGC CGA CTC C-3’

WNT7b

sense 5’-GGG ACT ATG AAC CGG AAA GC-3’

antisense 5’-TGC GGA ACT GAA ACT GAC AC-3’

WNT5b

sense 5’-TCT GAC AGA CGC CAA CTC CT-3’

antisense 5’-GCA TTC CTT GAT GCC AGT CT-3’

WNT2b

sense 5’-AGA CAT CAT GCG TTC AGT GG-3’

antisense 5’-TCA CAG CTG CAC ACA CTC AG-3’

LRP5

sense 5’-CTC TCC GGG GAC ACT CTG TA-3’

antisense 5’-CTC CTG CCT TAC ACG TCC TG-3’

LRP6

sense 5’- ACA GAC ACT GGC ACT GAT CG-3’

antisense 5’-GTT TTG GCA TCT CCC CAG TA -3’

TCF1

sense 5’- GAG GAG CAG GAC GAC AAG AG-3’

antisense 5’-GCA GAT TGA AGG CGG AGT AG -3’

TCF3

sense 5’-CGG TCC TCT CCT CAC ATG GT -3’

antisense 5’-TGC CAC TTT CTT CCA AGG AT -3’

TCF4

sense 5’-CAT ATG GTC CCA CCA CAT CA -3’

antisense 5’-TGC TCT TCT CTG GAC AGT GC -3’

LEF1

sense 5’-TCA TAT GAT TCC CGG TCC TC -3’

antisense 5’- CCT TCT GCC AAG AAT CTG GT-3’

β-ACTIN

sense 5’-GCA TTG TTA CAG GAA GTC CCT TGC C -3’

antisense 5’- ACC CAC TCC CAG GGA GAC CAA AAG-3’

**Q-PCR oligonucleotides:**

Axin2

sense 5’-GCC AAT GGC CAA GTG TCT CT-3’

antisense 5’- GCGTCATCTCCTTGGGCA-3’

Lef1

sense 5’- AGTGCAGCTATCAACCAGATCCT-3’

antisense 5’- TTTCCGTGCTAGTTCATAGTATTTGG-3’

MMP7

sense 5’- GGAGATGCTCACTTTGACAAGGA-3’

antisense 5’- ATTCATGGGTGGCAGCAAAC-3’

Cyclin D1

sense 5’- CGGCCCGAGGAGCTG-3’

antisense 5’- GGCCAGGTTCCACTTGAGC-3’

Dkk2

sense 5’-TCG CCA CTT CTG GAC CAA AA-3’

antisense 5’-TGC ACA GTC ACA CCT CTG GAA A-3’

Dkk3

sense 5’-CGC TGT TGC TAG AAA CGC TGT-3’

antisense 5’-TCC CCC AAA TCC ATC ATG AG-3’

**Cleaved caspase 3 immunostaining and FACS**

Cleaved caspase 3 was detected using a rabbit anti-cleaved caspase 3 antibody (Cell Signaling technologies). FACS analysis was performed using the same antibody following the manufacturer’s protocol.

# Cyclopamine treatment

The Hedgehog signaling inhibitor Cyclopamine (Toronto Research Chemicals) was used at a concentration of 10μM in cell medium. The culture media was changed every 48hrs. The pictures showing cell morphology were taken with a Nikon inverted microscope.

**References:**

1. Segara D, Biankin AV, Kench JG*, et al.* Expression of HOXB2, a retinoic acid signaling target in pancreatic cancer and pancreatic intraepithelial neoplasia. Clin Cancer Res 2005;11:3587-96.

2. Biankin AV, Morey AL, Lee C-S*, et al.* *DPC4*/Smad4 expression and outcome in pancreatic ductal adenocarcinoma. J Clin Oncol 2002;20:4531-4542.

3. Skalicky DA, Kench JG, Segara D*, et al.* Cyclin E expression and outcome in pancreatic ductal adenocarcinoma. Cancer Epidemiol Biomarkers Prev 2006;15:1941-7.
